# Supplementary material for: Culture-enriched metagenomic sequencing reveals within-patient diversity and transmission of vancomycin-resistant Enterococcus faecium
Source: Microb Genom. 2026 Jul 3;12(7):001778. doi: 10.1099/mgen.0.001778 (PMC13331451; doi:10.1099/mgen.0.001778)
Supplement: Supplementary Material 1. [file mgen-12-01778-s001.pdf]

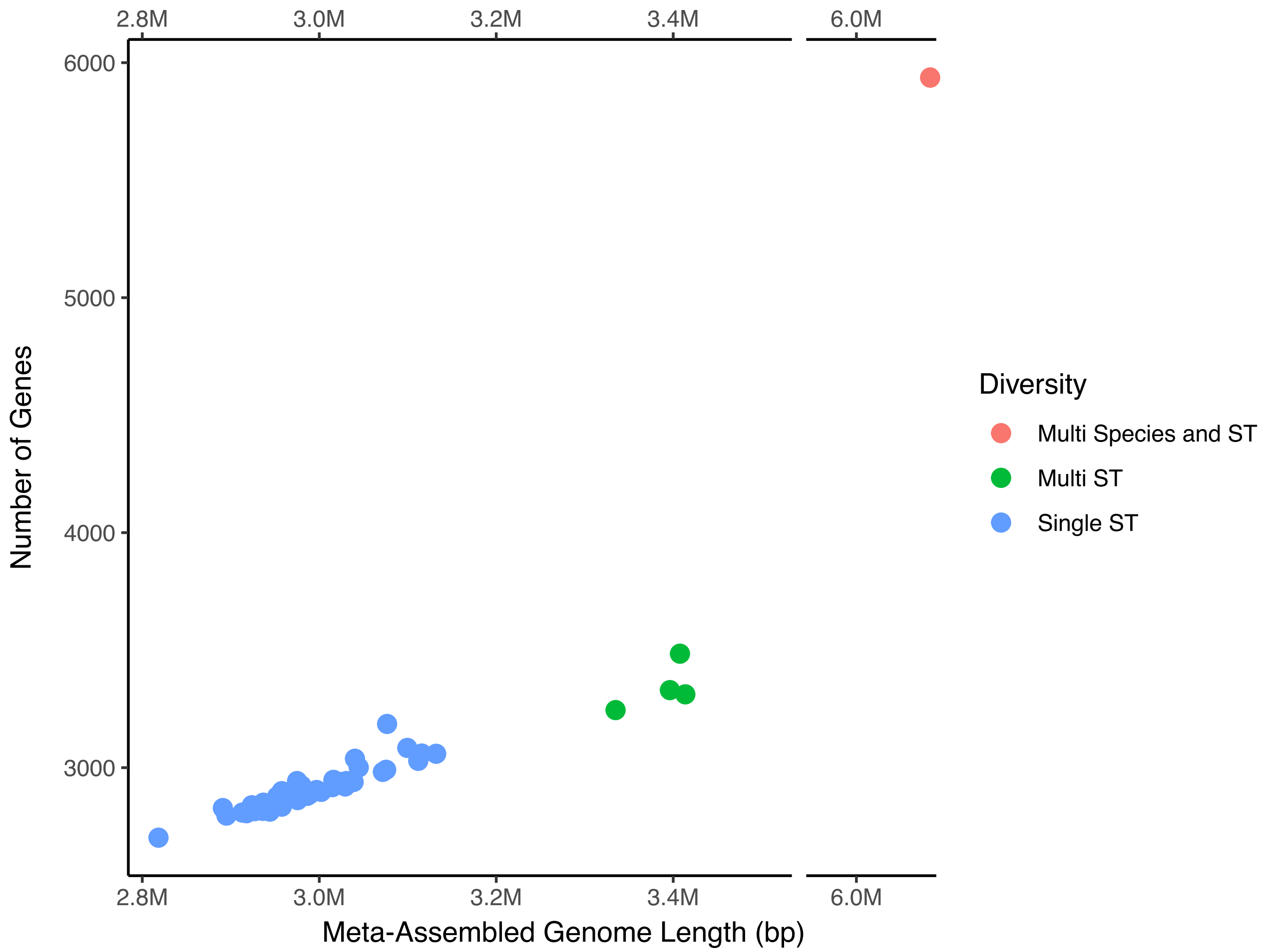

Figure S1. Populations with greater diversity show larger metagenome-assembled genomes and greater gene content. Blood and gastrointestinal tract populations were assembled using SPAdes with the --meta flag to generate metagenome-assembled genomes (MAGs). Gene counts were tabulated using Prokka. Each point represents one population, colored by within-sample diversity: multi-species and multi-sequence type (ST) populations (red), multi-ST populations (green), and single-ST populations (blue).

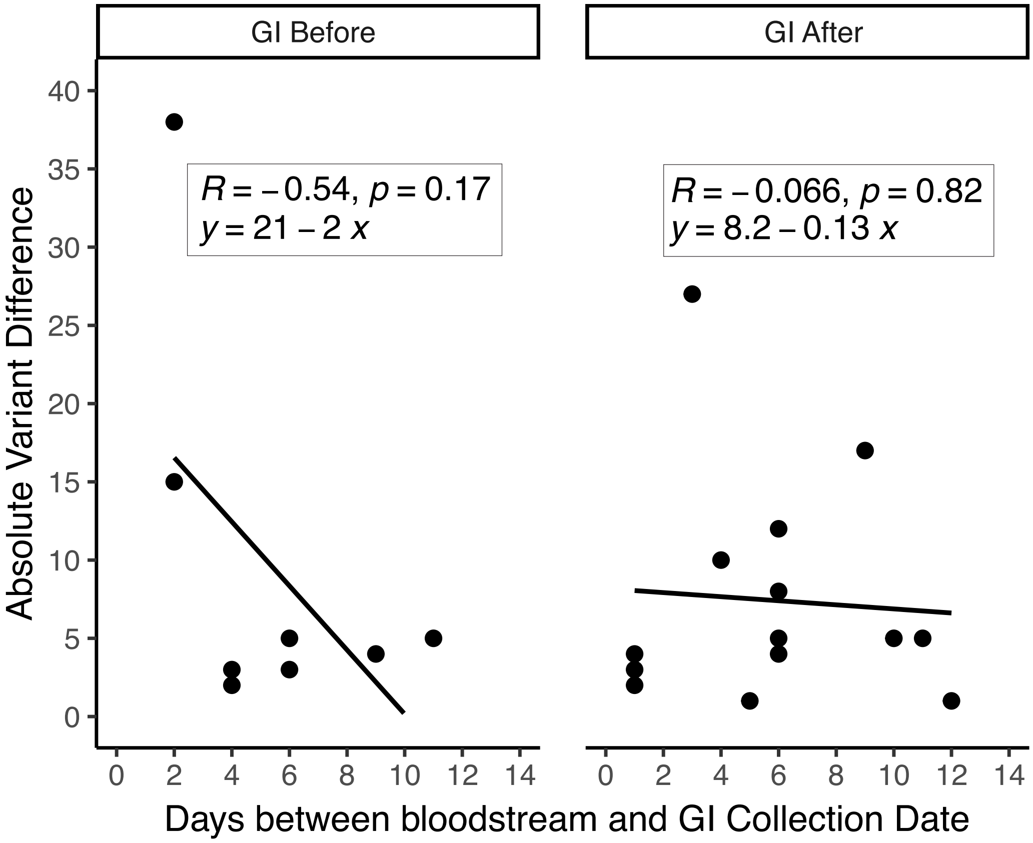

Figure S2. Association between sampling timeframe and variant diversity. Linear regression comparing the absolute variant differences between GI tract and bloodstream populations versus days between sampling for 22 patients with matching single-strain populations where the GI tract sample was collected either before or after the bloodstream sample. Patients with GI tract and bloodstream samples collected on the same day were excluded from analysis.
